# Editorial: Experiences of mental health promotion and suicide prevention

**DOI:** 10.3389/fpsyg.2025.1572859

**Published:** 2025-04-15

**Authors:** Santiago Gascón-Santos, Carla M. Santos de Carvalho, Adrián Alacreu-Crespo, Jorge L. Ordóñez-Carrasco

**Affiliations:** ^1^Department of Psychology and Sociology, University of Zaragoza, Zaragoza, Spain; ^2^Department of Social Psychology, University of Coimbra, Coimbra, Portugal

**Keywords:** mental health, promotion, prevention, experiences, suicide prevention, mental disorders

The increasing prevalence of mental disorders and their strong association with suicidal behaviors (Bae et al., 2022) have not only strained health systems around the world, but have also underscored the urgent need for innovative approaches to mental health promotion, early detection and suicide prevention, and the promotion of experiences aimed at detection, provision of support, and promotion of wellbeing.

Mental health results from a complex interaction of biological, psychological, social, and environmental variables (Turecki et al., 2019) that affect each age group differently. Concretely, in school environments bullying is a risk factor for psychological disorders. Wen et al. analyzed its effects on the risk of self-harm (Plener et al., 2015) and the mediation of variables such as alexithymia and rumination (Borrill et al., 2009), offering new perspectives for prevention (Wilkinson et al., 2011).

Kwon addresses the congruence between one's own values and those of the organization in young people, who show particular sensitivity to fairness (Moon et al., 2024) and to the fact that injustice is associated with increased vulnerability to anger and Hwa-byung syndrome (Hong and Hong, 2023). Factors associated with suicidal ideation differed by age, but depression and stress were risk factors at all ages (Hwang et al., 2024). Liu et al., explore how negative life events contribute to suicidal ideation. They attended to the mediating role of entity, whereas meaning in life moderated the likelihood of suicide (Song et al., 2022). This variable was related to quality of life and physical and mental health (Kim et al., 2019). Qifei et al. found that resilience mediated the effect of exercise on feelings of safety, while exercise increased feelings of safety through the resilience factor (Piestrzyński et al., 2021), improving memory, mood, and physical and mental health (Al-Qahtani et al., 2018). A controlled study on the effectiveness of a resilience-based intervention in at-risk adolescents Llistosella et al. is also included, finding that it contributed to fostering positive development, reinforcing the potential of multicomponent interventions. This life stage presents greater vulnerability to developing mental health problems (Ryu and Fan, 2023). Conceptualizing resilience as a dynamic process, they confirm the association between individual traits, context and social variables.

Outside the field of education, there are experiences of mental health in the workplace. Lee and Lee studied the relationship between stress, mental health and productivity. 26.2% of the workers had high levels of stress, with job control and job demands being the highest sub-factors. Stress resulting from organizational conditions led to dissatisfaction, absenteeism, decreased productivity, accidents, cardiovascular diseases, depression, anxiety, etc. (Kim and Kang, 2010). On the other hand, Dobešová Cakirpaloglu et al. confirm that stress negatively affects the performance, mental health and wellbeing of healthcare workers. Emotional exhaustion and stress were associated with feelings of anxiety and depression, while self-fulfillment mitigated depression and promoted wellbeing (Taranu et al., 2022). Conflict of values may lead employees to act inconsistently with their roles and promote burnout (Rotenstein et al., 2023). Mental or physical exhaustion was associated with prolonged stress and inability to choose effective coping strategies (Edward and Hercelinskyj, 2007).

In this line, a study on symptomatology associated with aggression suffered by health professionals is presented (Gascón-Santos et al.), 57.5% had suffered aggression in the last year (threats, insults and physical aggression). This group presented more psychological symptoms: such as re-experiencing, avoidance, distancing, emotional, or cognitive alterations and hypervigilance. A relationship was found between aggression and burnout. Previous research concluded that violence was common in the emergency and psychiatric services (Gascón et al., 2009), but in recent decades there has been a collapse in primary care, creating the conditions for these episodes to occur (Moleras-Serra et al., 2023).

Mental health and suicide are being studied through communication processes and the inappropriate use of social networks, Arik et al. present a qualitative study on media consumption habits and their relationship with suicidal ideation. Four types of factors emerged: psychological, familial, sociocultural and network-related. Most of those who had made a suicide attempt came from dysfunctional families with experiences of violence. As psychological factors they found problems of anger, hopelessness and maladjustment. Most of them spent much of their time interacting with content that produced adverse emotional states. Social media have become a tool for feeling recognized in virtual communities and their misuse can contribute to normalizing suicidal thoughts (Balt et al., 2023). But these platforms can be used as a preventive measure for those who are facing integration problems and are contemplating suicide (Cheng et al., 2015).

Finally, Myrick and Willoughby highlight the impact that public figures acknowledging a mental disorder can have. Such disclosures may prompt the public to reconsider their health behaviors. Such a response differs according to age groups and whether the celebrity is from the world of entertainment, sport or politics. In the latter case, reactions varied according to the ideology of the politician. In any case, the search for information related to the disease increased. They conclude that news about mental illness could be used to provide accurate and useful information about ways to help (Kresovich and Noar, 2020).
